# A Swedish evaluation of an educational intervention in person-centred communication for nursing assistants in home care for older adults: the ACTION programme

**DOI:** 10.1186/s12877-026-07507-6

**Published:** 2026-04-18

**Authors:** Birgitta Kerstis, Daniel Lindberg, Tanja Gustafsson, Jessica Höglander

**Affiliations:** 1 Department of Health Sciences, Innovation and Design , Mälardalen University, Västerås, SE-721 23 Sweden; 2 Department of Behaviour, Law and Social Science, Division of Social Work, Örebro University, Örebro, Sweden; 3 Faculty of Caring Science, Work Life and Social Welfare, University of Borås, Borås, Sweden

**Keywords:** Communication, Competence, Development, Education, Home care, Intervention, Nursing assistant, Older adults, Person-centred, Training

## Abstract

**Background:**

Home Care Services for older adults vary widely across countries regarding how they are structured, organised, and funded. Contextual factors such as workload, staffing challenges, and variability in educational backgrounds may influence participation in communication training. Previous evaluations of the ACTION programme (A person-centred CommunicaTION programme for nursing assistants (NAs) in home care for older adults) have shown promising feasibility and acceptability, providing the basis for the current scaled-up evaluation. This study aimed to evaluate the impact of ACTION on NAs in Swedish home care, using a mixed-methods cluster design.

**Methods:**

This study used a convergent mixed-methods cluster quasi-experimental design Data were collected from both intervention and control groups using pre- and post-intervention surveys assessing person-centred care, empathy, job satisfaction, and communication self-efficacy. The intervention group also completed a programme evaluation with open-ended questions upon completing the training modules.

**Results:**

Quantitative results showed no statistically significant differences between groups after cluster-corrected analysis, while qualitative findings indicated increased awareness and reflection regarding communication.

These findings highlight the importance of considering contextual factors and providing ongoing support when implementing person-centred communication interventions in elder care. The evaluation shows both strengths and methodological limitations. While some positive qualitative changes were observed, the findings emphasise the need to include the perspectives of older adults and their relatives in future research.

**Conclusions:**

While quantitative effects were limited, the training programme contributed to meaningful qualitative improvements in communication awareness. Longer follow up and larger samples are needed to determine measurable effects.

**Trial registration:**

ISRCTN64890826. Registered 10 January 2022, https://www.isrctn.com/ISRCTN64890826.

**Supplementary Information:**

The online version contains supplementary material available at 10.1186/s12877-026-07507-6.

## Background

The study introduces the ACTION programme, A person-centred CommunicaTION programme for nursing assistants (NAs) in home care for older adults. Home care services for older adults differ internationally in organisation, structure, and delivery, yet communication remains central to high-quality care across settings. In Sweden, NAs constitute one of the largest professional groups within home care and provide most direct support, including personal care, assistance with daily activities, and basic health-related tasks [1]. They perform a variety of tasks that require not only practical nursing skills but also interpersonal and communication competencies. NAs assist with daily living activities such as personal hygiene, dressing, and mobility, and administer basic medical care, such as medication management and wound dressing. They are also expected to respect each older adult’s dignity and individuality while supporting their emotional and psychological well-being. However, NAs report facing challenges due to time constraints, rigid schedules, and lack of training [2].

Older adults receiving home care frequently present with frailty, cognitive impairment, multimorbidity, and a combination of physical, emotional, and existential needs. These needs are often subtly expressed [3], requiring attentive and responsive communication skills from NAs. Previous research has shown that communication training can enhance knowledge, improve communication skills, and enhance job satisfaction among healthcare professionals [4, 5]. However, evidence specific to NAs working in Swedish home care contexts remain limited. The home care environment is also characterised by conflicting demands from the healthcare system, time pressure and organisational constraints [2, 6], further complicating opportunities for reflection, learning, and implementing communication strategies.

Person-centred care (PCC) emphasises understanding and responding to each individual’s preferences, values, and needs [7]. In McCormack and McCance’s framework, person-centred communication is not separate from PCC, it is the primary means through which PCC is achieved [8]. Person-centred communication involves relational processes that support understanding of the individual, autonomy, shared decision-making, trust, and wellbeing. These processes involve recognising everyone as a unique person, inviting their participation in care, and responding attentively to both verbal and non-verbal cues. Person-centred communication also includes validating the older person’s emotions, listening actively, and creating space for meaningful dialogue. Such communication practices have been shown to strengthen the sense of dignity, connectedness, and partnership in care relationships, and are essential for supporting older adults’ involvement in decision-making and self-management [9]. Complementing this perspective, person-centred communication has been shown to rest on three core attributes: recognising, inviting, and involving the older adult [10]. These involve acknowledging the person’s emotions and perspectives, creating space for their participation, and engaging them in care-related dialogue, supported by healthcare professionals’ attentiveness and responsiveness. There is, however, still a gap between the theoretical understanding and the practical implementation of PCC, largely due to organisational barriers such as low staffing levels, limited caregiver motivation, insufficient recognition of efforts, and a lack of opportunities for continuous professional development [7].

In response to these challenges, the ACTION programme was developed. This web-based educational training intervention was co-created by researchers, managers, and NAs using a participatory design approach. The training programme aims to enhance the competence of NAs in person-centred communication when providing home care for older adults. It has been pilot-tested and evaluated in studies that provide insights into its implementation, feasibility, and outcomes [2, 11, 12]. The training programme was initiated in 2017 with a small-scale intervention [11]. The evaluations indicated that communication training can enhance communication skills and improve job satisfaction by fostering a sense of accomplishment and connection with older adults. They also highlighted areas in need of refinement, while demonstrating promising results that form the foundation for the development of this scaled-up intervention. The training programme is mainly web-based and integrates essential components of person-centred communication, including attentiveness, empathy, active listening, and the ability to recognise and respond to emotional cues. Its pedagogical design is grounded in self-directed and reflective learning principles and is delivered through six sequential modules comprising web-based lectures, quizzes, reflection tasks, and group supervision. The training modules progress stepwise over six weeks: Week (1) introduces the principles of person-centred communication; Week (2) focuses on verbal and non-verbal communication; Week (3) deepens competence in being present and listening; Week (4) centres on “good conversations” in everyday home care situations; Week (5) includes group reflection on communication and behaviours, allowing guided reflection on challenges and advanced communication strategies; and Week (6) focuses on person-centred communication in complex situations, such as end-of-life conversations and handling aggressiveness, and different contexts [13]. The programme encourages integrating personal experiences into learning and applying knowledge daily [2, 11, 12]. To gain a deeper insight into the significance of competence development in communication, this study aimed to evaluate the impact of ACTION on NAs in Swedish home care.

## Methods

### Design

This study is a part of a larger research initiative; the ACTION programme [13] and used a convergent mixed-methods cluster quasi-experimental design [14]. Ethical approval was obtained from the Swedish Ethical Review Authority (Dnr 2021–05233), in accordance with the principles of the Declaration of Helsinki [15].

### Setting and samples

Fourteen home care team managers in Mid-Sweden were contacted by one of the co-authors regarding participation in the ACTION programme and eleven teams agreed to participate and were randomly allocated at cluster level to either the intervention group or control group. The randomisation was carried out by an independent researcher who was not involved in recruitment, data collection, or delivery of the intervention. Because allocation occurred at the team level and managers were aware of their group assignment, allocation concealment was not possible. All eligible NAs with ongoing, non-temporary employment were invited to participate in the study. NAs with temporary employment (i.e., hired on an hourly or casual basis) were offered the opportunity to take part in the training but were not included in the research. This exclusion was based on ethical considerations, aiming to reduce the risk that individuals without permanent positions might feel pressured to participate.

**Fig. 1:**
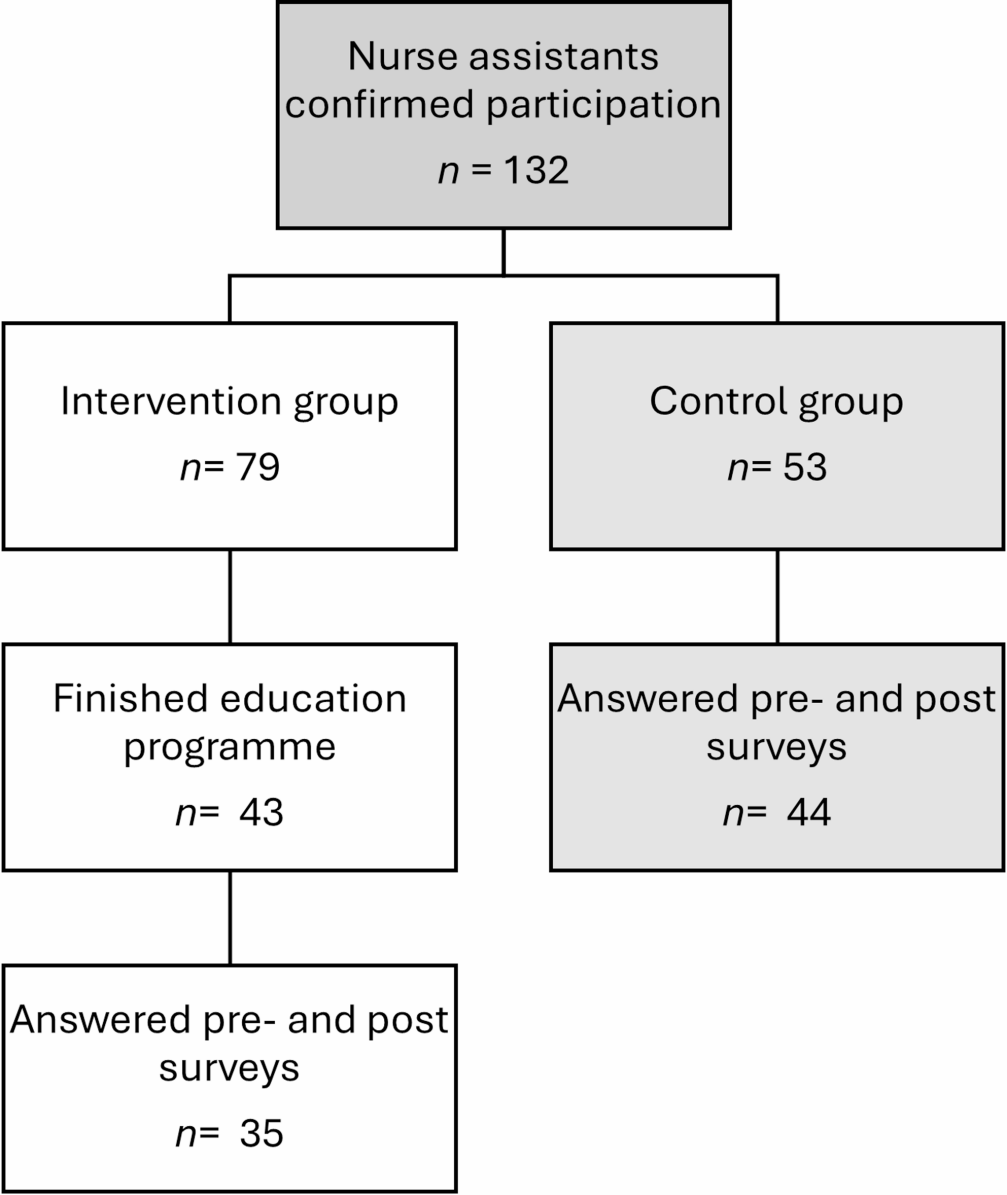
Flowchart of participation in the intervention and control group

A total of 132 NAs agreed to participate, resulting in 79 NAs in the intervention group (70 women, nine men) and 53 in the control group (43 women, 10 men). Although 132 NAs initially consented to participate, attrition occurred due to workload and time constraints, resulting in varying numbers of complete pre- and post-survey responses in the intervention and control group (see Fig. 1). These differences, along with missing data, were addressed in the statistical analysis using cluster-adjusted Generalized Estimating Equations (GEE) models. Additionally, not all enrolled NAs in the intervention group completed the training programme, primarily due to demanding working conditions. Approximately half (*n* = 43) completed all training modules and the evaluation form.

The possibility of spill-over between intervention and control teams was considered. Teams were organisationally separate and scheduled independently, reducing the risk of communication or knowledge transfer across clusters. Nevertheless, minimal contamination cannot be entirely excluded and is considered in the study’s limitations. All analyses treat the team as the unit of clustering, consistent with the allocation procedure. Therefore, team membership is included as the clustering variable in all GEE models. A description of the sample is presented in Table 1.

**Table 1:** Overview of participation in the intervention and control group

Group		Intervention *n* = 43	Control *n* = 44	Sign	
Female, *n* (%)	33 (76.7)		34 (77.3)		NS ^b^
Age, years; mean, SD (min- max)	43.2, 10.5 (26–61)		42.4, 13.6 (19–64)		NS ^a^
Working experience in care of older adults, months; mean, SD (min-max)	14.2, 8.9 (2–36)		16.5, 11.4 (1–40)		NS ^a^
Second language *n* (%)	10 (23.3)		10 (22.7)		NS ^b^

*NS* Not Significant

^a^ *t*-tests, ^b^ chi-square tests

### Data collection

To evaluate the effect of the intervention on NAs’ communication, data were collected through surveys using validated instruments, as well as responses from an evaluation of the training programme. The validated instruments included questions related to PCC [16], empathy [17], job satisfaction [18], and self-efficacy in communication skills [19]. Furthermore, the surveys included demographic questions about sex, age, native language, and work experience in the care of older adults. The intervention group completed a baseline survey before the intervention and another survey six to eight weeks later. The control group completed the same pre- and post-surveys as the intervention group [13]. At the end of the training programme, the intervention group completed an evaluation. This evaluation consisted of open-ended questions and was administered as a final assignment in the last training module. The questions included were: 1*) What do you think has been important and interesting about the communication training programme? (2) What new knowledge and ideas have you gained? (3) How do you think your way of communicating might have been affected after the training programme?* and *(4) Is there anything you felt was missing or that could be improved in the training programme?*

All data collection and participation in the ACTION programme took place during the NAs’ regular work hours, with time specifically allocated within their schedules to participate.

### Measures

#### Person-centred care assessment tool

The Person-Centred Care Assessment Tool (P-CAT) measures to what extent staff members rate the care provided as being person-centred. It uses 13 items, rated on a five-point Likert-type scale ranging from 1 (disagree completely) to 5 (agree completely), with scores from 13 to 65, where higher values indicate a higher degree of self-scored person-centredness [16]. Cronbach’s alpha was 0.642 and 0.639 across the two measurements.

#### Jefferson scale of empathy

The Jefferson Scale of Empathy (JSE) measures empathy in the context of health professions education and patient care and is designed for use with health professions students and practitioners [17]. The 20 items are on a five-point Likert-type scale ranging from 1 (disagree) to 7 (Strongly Agree), with scores from 20 to 140, where higher values indicate a higher degree of self-scored empathy (Cronbach’s alpha was 0.689 for survey 1, and 0.808 for survey 2). The scale consists of three factors: I *Perspective-taking* (ten items) (Cronbach’s alpha was 0.718 for survey 1, and 0.804 for survey 2): II *Compassionate care* (eight items) (Cronbach’s alpha was 0.537 for survey 1, and 0.704 for survey 2), and III: *Walking in the patient’s shoes* (two items) (Cronbach’s alpha was 0.641 for survey 1, and 0.659 for survey 2). 

#### Measure of job satisfaction

Job satisfaction measures to what extent staff rate job satisfaction, with 37 items on a five-point Likert-type scale ranging from 1 (Very displeased) to 5 (Very pleased), with scores from 1 to 185, where higher values indicate a higher degree of job satisfaction [18]. Cronbach’s alpha was 0.833 for survey 1, and 0.835 for survey 2. The question *My clinical grading* was excluded from the survey because it was not relevant to this group. The scale consists of five factors: I *Personal satisfaction* (ten items) (Cronbach’s alpha was 0.405 for survey 1, and 0.346 for survey 2), II: *Satisfaction with workload* (seven items) (Cronbach’s alpha was 0.358 for survey 1, and 0.137 for survey 2), III: *Satisfaction with professional Support* (nine items) (Cronbach’s alpha was 0.690 for survey 1, and 0.675 for survey 2), IV: *Satisfaction with pay and prospects* (eight items) (Cronbach’s alpha was 0.582 for survey 1, and 0.792 for survey 2), and V; *Satisfaction with training* (three items) (Cronbach’s alpha was 0.332 for survey 1, and 0.681 for survey 2).

#### Self-efficacy questionnaire

The Self-Efficacy Questionnaire (SE-12) measures the clinical communication skills of healthcare professionals [19]. The 12 items are on a 10-point Likert-type scale ranging from 1 (very uncertain) to 10 (very certain), with scores from 12 to 120, where higher values indicate a higher degree of self-scored clinical communication skills. Cronbach’s alpha was 0.862 and 0.878 across the two measurements.

### Analyses

#### Quantitative analyses

Descriptive statistics were analysed, using frequencies and percentages for categorical variables, and means and standard deviations (SD) for continuous variables. Independent sample t-tests were employed to assess group differences in continuous variables, while chi-square tests were utilised for categorical variables. Age and experience in the care of older adults were analysed using *t*-tests, whereas gender and second language status were compared through chi-square tests. GEE was conducted with team as the clustering variable to account for intracluster correlation. The primary outcome was the P-CAT total score; secondary outcomes were the JSE total score, the job satisfaction total score, and the SE-12 total score. No subscale scores from the Job Satisfaction scale were analysed due to low internal consistency. The group × time interaction term was used to estimate the intervention effect. Missing data was handled within the GEE framework without imputation. Attrition remained between 5 and 10% across groups, with no systematic missing-data patterns detected. Effect sizes and 95% confidence intervals will be reported for all outcomes. Because several subscales demonstrated unacceptably low internal consistency (α < 0.50), only the total job satisfaction score was used in all statistical analyses; subscale scores were not interpreted. All instruments used in the study have previously been validated in Swedish samples and shown acceptable psychometric properties. In the present study, internal consistency ranged from α = 0.64 to 0.88 across measures, except for certain job satisfaction subscales. All tests were two-tailed, and statistical significance was set at *p* ≤ .05. Two of the authors (BK and DL) conducted the main part of the quantitative analysis, while all authors contributed to discussions throughout the analytic process to ensure rigour. All analyses were conducted using IBM SPSS Statistics (Version 28.0; IBM SPSS, Armonk, NY, USA).

#### Qualitative analysis

A total 43 evaluations were analysed. Answers from the evaluation form were analysed following an inductive content analysis [20]. The texts were read several times to give the authors a thorough understanding of their content. Secondly, meaning units, such as words, sentences or sections related to the study aim, were identified in the text. With the analysis, meaning-bearing units were re-read to obtain a sense of the whole and become immersed in the data. Thereafter, the data was coded, grouped into categories and abstracted. The codes were then compared to identify similarities and differences, leading to the emergence of seven subcategories and three generic categories, which were ultimately synthesised into a main category (see Table 2) [20]. Two of the authors (JH and TG) conducted the main part of the qualitative analysis, while all authors contributed to discussions throughout the analytic process to ensure rigour.

**Table 2:** Examples of the abstraction process

Subcategories	Generic categories	Main category
Good communication Non-verbal	Inclusive communication	Experiences of communication training
Relationships Trust	Building relationships	
Questions Listening Understanding	Active listening	

## Results

The results were divided into two main sections: statistical answers from the surveys and qualitative answers from the evaluation forms.

### Results from the surveys

There were no significant differences in changes over time between the intervention and control groups for P-CAT and empathy scores. Similarly, for self-efficacy, neither time nor group effects were significant, and no evidence of differential change was observed. Regarding work-related outcomes, there was a trend suggesting greater improvement in the intervention group compared to the control group, but this trend did not reach statistical significance, as the confidence interval included zero (Table 3).

**Table 3:** Generalized Estimating Equations GEE models estimates of intervention effects over time across outcome measures p*erson-centred care assessment tool*,* empathy*,* job satisfaction*, and c*linical communication skills*

Outcome	B (Time x Team)	SE	95% CI Lower	95% CI Upper	Wald x^2^	*p*-value
P-CAT	0.17	1.34	-2.45	2.80	0.02	0.897
Empathy	0.25	2.55	-4.75	5.26	0.01	0.921
Job satisfaction	5.53	2.94	-0.23	11.30	3.54	0.060
Self-Efficacy	1.43	1.44	-1.39	4.25	0.99	0.320

B (Time × Team) represents the coefficient for the interaction between time and the clustering variable *team* in the GEE model, accounting for intracluster correlation. SE denotes the standard error of the estimate. The 95% CI shows the lower and upper confidence bounds. The Wald χ² value tests the statistical significance of the parameter estimate

*P-CAT* Person-Centred Care Assessment Tool

### Results from the evaluation forms

The qualitative findings from the evaluation form completed by the intervention group are presented below. One main category *Experiences of communication training* and three generic categories: *Awareness of the importance of communication*,* Value of repeating and reinforcing knowledge*,* and Strengths and areas for development in the training programme* emerged from the analysis.

### Experiences of communication training

The evaluations from the intervention group following the training programme indicated that the training was perceived as valuable and had increased the NAs’ awareness of the impact of communication and its significance in their interactions. In their evaluations, the NAs described a deeper understanding of how communication shapes care encounters and highlighted how the repetition of previous knowledge served as a helpful reinforcement. Additionally, they provided constructive feedback on how the design of the educational training could be further improved.

#### Awareness of the importance of communication

This category illustrates how the intervention strengthened NAs’ ability to reflect on and articulate communication as an intentional and professional skill, beyond routine interactions. Communication was described as a fundamental and important part of care, and the ACTION programme had helped increase the NAs’ awareness of the importance of good communication.

*Communication is important*,* that has always been known*,* but through the course [ACTION programme]*,* I have realised how much more and in everything we do*,* say*,* and don’t say*,* we are communicating (NA2).*

NAs described an increased awareness of communication as essential for creating trust, confidence, and establishing good relationships with the older adult. They also emphasised communication as crucial for enhancing their understanding of the older adult. One example of how to increase their understanding was through active listening.

*Communication training shows how important it is to listen actively and affirm the older adult (NA8).*

Many of the NAs said that the training programme had reminded them of the importance of taking the time to listen and not be hurried. Several described how the training increased their awareness of the importance of actively demonstrating that they were listening, for example through non-verbal behaviours such as tone of voice, posture, and facial expressions.

Others described how the training reminded them of the value of asking questions during their visits, how it encouraged them to do so intentionally, including questions that might feel difficult or uncomfortable. They also reflected on the importance of allowing silence during conversations, recognising it as a meaningful and valuable part of communication.

#### Value of repeating knowledge

The training was described as a valuable opportunity to revisit and reflect on existing knowledge, as well as to identify knowledge gaps. Rather than introducing entirely new concepts, it served as a structured reminder of communication principles that many NAs already applied in their daily work, often without thinking about it.

*Some things I already knew*,* but I learned a lot as well. I will carry this with me both in my personal and professional life (NA34).*

The reflective elements of the training programme encouraged them to view their communication practices through a more intentional and professional lens. Several NAs expressed that the training helped them recognise and articulate what they already did well, while also identifying areas where their communication could be improved.

*I thought I knew a lot about communication before*,* but I was wrong. I feel that I have grown from this education (NA13).*

#### Strengths and areas for development in the training programme

The NAs generally expressed appreciation for the structure and content of the training. Elements such as recorded lectures, group reflections, and podcasts were highlighted as particularly useful and relevant. The inclusion of real-life scenarios and practical strategies was seen as helpful for translating communication theory into everyday practice. A podcast focusing on challenging communication and interactions with people with dementia, was especially noted as interesting and valuable.

*I have found it interesting to listen to the podcasts. The latest one was particularly valuable*,* discussing how to listen and talk to those with dementia or who have other illnesses that can make it difficult to connect or find topics of conversation*,* and how to remain calm when talking to them (NA25).*

The training programme was also described as providing useful new tools and ideas for communication. The NAs also shared concrete examples of communication strategies they had adopted, including mirroring, maintaining presence, and using silence more intentionally.

The NAs also provided suggestions and identified areas for improvement. Although the training module specifically addressed communication strategies for managing aggressive or challenging situations, it was suggested that additional content on this topic would be beneficial. Furthermore, there was an expressed need for newly hired employees and substitutes to have ongoing access to the training programme, as it is considered essential for enhancing their communicative skills and interactions with the older adults in their care.

*I found it interesting to listen to this [training programme]*,* and you can always learn something new… It would be great for newly hired nursing staff and substitutes to listen to this because I believe it could help many who do not have much experience in this field (NA3).*

Finally, it was noted that learning how the digital platform functions could be challenging, especially for those with limited technical skills.

Overall, the qualitative findings indicate that the ACTION programme supported reflective learning processes and enhanced NAs’ consciousness of communication as a central component of person-centred care. These insights provide important contextual understanding of the mechanisms through which the intervention may influence practice, even when quantitative effects are limited.

## Discussion

This study evaluated the effects of a person-centred communication intervention for NAs working in Swedish home care. It is important to distinguish between person-centred care (PCC), which refers to the overall philosophy of tailoring care to the individual, and person-centred communication, which was the specific focus of the ACTION programme. The intervention specifically targeted communication behaviours rather than broader organisational aspects of PCC, distinguishing person-centred communication as a core interpersonal skill fundamental to high-quality care. While the cluster-adjusted quantitative analyses showed no statistically significant changes over time or between groups, the qualitative findings indicated meaningful developments in NAs’ awareness, intentionality, and reflective capacity.

The mixed-methods design provides important insights by showing how different forms of evidence complement one another [14]. The qualitative findings help contextualise the lack of quantitative effects. Although no measurable quantitative improvements were detected, the qualitative results illustrated early cognitive and attitudinal change, including greater attention to non-verbal communication, active listening, and relational awareness. Such cognitive and reflective shifts often occur before observable behavioural changes and align with previous research showing that communication training can lead to improvements in self-efficacy and perceived communication skills among healthcare providers, while behavioural effects may require more time to develop [4, 5].

The absence of significant quantitative effects must be interpreted considering contextual and methodological constraints. The cluster design and attrition reduced statistical power, making it difficult to detect small effects. Many NAs reported high confidence in their communication abilities at baseline, which may have limited the potential for measurable improvement due to a ceiling effect. However, this cannot be confirmed without distributional analysis and should be carefully interpreted. The short follow-up period of six to eight weeks may also have limited the opportunity to observe changes, as behavioural integration of new communication strategies often requires sustained organisational support and time for practice [9]. In addition, variation in attendance and limited follow-up may have affected the fidelity of the intervention and its practical implementation.

The qualitative findings represent participants’ subjective perceptions and should be interpreted in relation to the non-significant quantitative findings. Nevertheless, they offer valuable insights into how the intervention facilitated learning. In particular, features such as repetition, structured reflection, and case-based examples appeared to support learning, knowledge development, and a deeper understanding of communication practices. The final qualitative category, which addressed strengths and areas for development in the training programme, contributes to the study’s aim by illustrating how structural and contextual aspects (e.g., access to digital support and time for competence development) influenced NAs’ opportunities to engage in person-centred communication. These reflections highlight the need to consider contextual and organisational conditions, such as workload, digital literacy, and staff turnover, when implementing educational interventions. Participants’ suggestions, including the need for more content on managing challenging situations and ensuring access to the training programme for newly hired staff, provide important considerations for future implementation of the ACTION programme. The NAs also described becoming more aware of their own body language, tone of voice, and the importance of asking questions, even those that might feel uncomfortable, to better understand the needs of older adults.

The broader organisational context also influenced the potential impact of the intervention. Time constraints and heavy workloads affected participation in both the study and the communication training programme. These observations align with earlier research describing barriers to person-centred practices in the care of older adults, such as insufficient resources, competing demands, and limited opportunities for continuous training [7, 9]. Addressing these organisational barriers is important to ensure that person-centred communication interventions can be fully effective in practice.

While this study focused primarily on NAs’ perspectives and self-reported communication, future research should incorporate the experiences of older adults and relatives to provide a more comprehensive understanding of relational outcomes. Longer follow-up, observational data, and larger samples are recommended to assess whether early gains in communication awareness and purposefulness result in measurable improvements in care. Qualitative methods proved valuable in capturing subtle changes that may not be immediately visible in quantitative data, highlighting the importance of including subjective evaluations when assessing the impact of communication training.

### Strength and limitations

A strength of this study is the mixed-methods cluster design, which enabled an in-depth understanding of how the ACTION programme was experienced in home care practice. The use of validated instruments and inclusion of qualitative reflections from NAs’ evaluations strengthened the credibility of the findings. However, several limitations must be considered. Participation attrition reduced the number of participants who completed both measurements, which limited the statistical power. Some job-satisfaction subscales showed low reliability and were therefore excluded from analysis, with only total scores used. The follow-up period of six to eight weeks may have been too short for measurable behavioural changes to appear. All outcomes were based on self-reported data, and no observational assessments or perspectives from older adults or relatives were included. Future studies should include their perspectives, as well as observational assessments. There was also a risk of contamination between intervention and control teams, as some informal communication or knowledge sharing could not be entirely prevented despite organisational separation. The findings reflect the Swedish home care context, where the education, roles, and responsibilities of NAs may differ from those in other countries, potentially limiting transferability. The trustworthiness of the results was supported by detailed descriptions of the participants, data collection procedures, and analyses.

## Conclusions

This mixed-methods cluster study found no statistically significant effects of the ACTION programme on quantitative measures of person-centred communication, empathy, self-efficacy, or job satisfaction. However, qualitative findings indicated that the training programme enhanced NAs’ awareness of communication and supported reflective learning, as expressed in their evaluations. These insights may reflect initial changes in thinking and attitudes to communication that require longer follow-up or organisational support to become measurable and visible in practice. Future studies should include older adults’ perspectives, longer intervention periods, and observational outcomes to better understand the impact of communication training in home care.

## Supplementary Information

Supplementary Material 1.

Supplementary Material 2.

## Data Availability

The datasets generated and/or analysed during the current study are not publicly available due to the sensitive nature of the data and ethical considerations.

## Abbreviations

ACTION: A person-centred communication programme
GEE: Generalized Estimating Equations
NA: Nursing assistant
PCC: Person-centred care
