# Supplementary material for: A Swedish evaluation of an educational intervention in person-centred communication for nursing assistants in home care for older adults: the ACTION programme
Source: BMC Geriatr. 2026 Apr 18;26:549. doi: 10.1186/s12877-026-07507-6 (PMC13091244; doi:10.1186/s12877-026-07507-6)
Supplement: Supplementary file 1 — Supplementary Material 1. [file 12877_2026_7507_MOESM1_ESM.pdf]

Jefferson
Philadelphia University +
Thomas Jefferson University

Center for Research
in Medical Education
and Health Care

1015 Walnut Street
Curtis Building, Suite 319
Philadelphia, PA 19107
T 215-955-9458
F 215-923-6939
jefferson.edu/crmehc

November 10, 2025

Jessica Höglander
Mälardalen University
Sweden

Dear Dr. Höglander,

This letter documents your permission to use the Jefferson Scale of Empathy in your research study confirmed via email on May 21,2021. With your agreement to all conditions stated in our previous emails, permission to administer 200 paper copies of the Swedish translation of the HP-version of the JSE with 200 health professionals was confirmed for the single not-for-profit study that you described, for a 12 month time period. I have sent a copy of the scale, the User's Guide and the scoring algorithm for completion complete your study.

The Jefferson copyright sign, printed at the bottom of the scale, must appear on any copy you use in your project, and proper credit should be given to the original source in any report. If the results of this project intend to be published, we ask that the statement be included, "The JSE was used in this study with permission from Thomas Jefferson University." Please send us a copy of any publication resulting from the study.

Also, per the conditions, reproducing the JSE in print, publications, or posting it on the web in its entirety is strictly forbidden without the permission of TJU. However, publishing up to 3 items is permissible for illustrative purposes. If any other researcher expresses interest in using the JSE, please have them contact us. Permission to use any version/translation of the JSE may be given only by us.

We wish you success with your research! Please keep us informed of your progress. If you have any questions, please feel free to contact me.

Good luck with your project!

Sincerely,

Shira Carroll
Empathy Project Coordinator
Center for Research in Medical Education & Health Care
empathy@jefferson.edu
